# Spread of Academic Success in a High School Social Network

**DOI:** 10.1371/journal.pone.0055944

**Published:** 2013-02-13

**Authors:** Deanna Blansky, Christina Kavanaugh, Cara Boothroyd, Brianna Benson, Julie Gallagher, John Endress, Hiroki Sayama

**Affiliations:** 1 Maine-Endwell High School, Endwell, New York, United States of America; 2 Binghamton University, State University of New York, Binghamton, New York, United States of America; Universitat Rovira i Virgili, Spain

## Abstract

Application of social network analysis to education has revealed how social network positions of K-12 students correlate with their behavior and academic achievements. However, no study has been conducted on how their social network influences their academic progress over time. Here we investigated correlations between high school students’ academic progress over one year and the social environment that surrounds them in their friendship network. We found that students whose friends’ average GPA (Grade Point Average) was greater (or less) than their own had a higher tendency toward increasing (or decreasing) their academic ranking over time, indicating social contagion of academic success taking place in their social network.

## Introduction

Application of social network analysis [1] to educational systems is a promising yet unexplored research area. A small number of studies investigated how the positions of K-12 students in their social network are correlated with their behavior and academic achievements. Farmer and Rodkin’s work [2] was among the earliest of its kind, in which they studied correlations between social network centralities of elementary school children and their behavioral traits, such as cooperativeness, leadership, popularity, athleticism and aggressiveness. More recently, Bishop [3] found, through statistical analysis of the Add Health data [4], that students in positions with higher centrality had significantly better grades than those with lower centrality. Similar correlations between academic achievements and centrality or popularity in social networks were also found in different data sets [5], [6], though it was also reported that the correlations depended significantly on ethnic and other social contexts [5].

These earlier findings naturally leads one to ask another question that may be more relevant to educators and students themselves: How does a student’s social network environment influence his or her academic progress *over time*? Unfortunately, none of the studies mentioned above provides a direct clue to this question, because their statistical analyses were all applied to static snapshots of students’ social networks and their grades, without any temporal changes taken into consideration.

To gain insight into the aforementioned question, we investigated, for the first time, the correlations between high school students’ academic progress over one year and the social environment that surrounds them in their friendship network. The students’ social network was reconstructed based on the results of an electronic survey asking them about their friendships, while the data about their academic progresses were obtained directly from their school’s official academic records. Our results showed that students whose friends’ average GPA (Grade Point Average) was greater (or less) than their own had a higher tendency toward increasing (or decreasing) their academic ranking over time, indicating social contagion of academic success taking place in their social network.

## Methods

### Ethics Statement

The study was approved by the Binghamton University Institutional Review Board with permission from the Maine-Endwell Central School District. All data were obtained with written informed consent reviewed by the Binghamton University Institutional Review Board (IRB). According to the research protocol reviewed and approved by the Binghamton University IRB, the researchers are not allowed to share the data with third parties outside the research team. Contact the corresponding author for more details.

We reconstructed a social network of the eleventh grade class at Maine-Endwell High School, Endwell, NY, USA, by developing an online survey and administering it on January 11, 2011. We received 160 responses (response rate: 92%), two of which were incomplete and therefore excluded from the statistical analysis (*N = *158). In the survey, the students were given a list of all the other students taking the survey, and asked to decide whether each of them were a best friend, a friend, an acquaintance, someone they did not know, or if they were related. The first three categories were used for social network reconstruction and analysis. Those self-reported friendships were represented as directed links in the reconstructed network.

The survey data were supplemented by data from the School’s student record database that contained information about GPA (at two time points: January 2011 and January 2012), attendance, disciplinary action, and gender for each student. All the data were anonymized and stored using randomized ID’s so that no personally identifiable information was kept in our records.

The GPA data were used to measure each student’s academic progress over the year. The original GPA distributions were highly skewed negatively, and there was an overall trend of GPAs moving upward over the year. We therefore transformed the raw GPA scores into academic rankings within the class. Rankings were calculated by subtracting the student’s position in the class from the total number of students, so that greater ranking values mean higher academic ranks. We then characterized the social environment surrounding a student by calculating the difference in ranking between each student’s self-reported neighbors’ average GPA and his/her own GPA (***x_i_***, where ***i*** is the type of the network: acquaintance, friend, or best friend). The student’s academic progress (***y***) was characterized by the increase or decrease of his/her academic ranking in the period spanning January 2011-January 2012.

## Results

**Figure 1:**
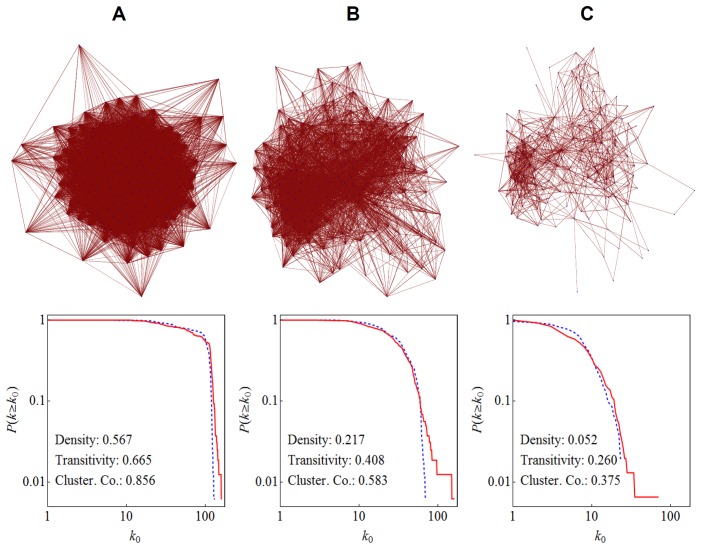
The social network in the junior class of Maine-Endwell High School at three different levels. Top: Visualizations of networks. Nodes (blue dots) represent individual students. Links (thin red lines) represent their friendships reconstructed from results of the online survey administered in January 2011. Directions of links are omitted for clarity. These plots show the entire network (i.e., there were no disconnected nodes or components in our data). Bottom: Complementary cumulative degree distributions and other network metrics. Red solid lines are for out-degrees, while blue dashed lines for in-degrees. Densities and transitivities were calculated using directed links, while average clustering coefficients were calculated after converting all the links into undirected ones. (A) A network made of all levels of links (acquaintances, friends, and best or close friends). (B) A network made of links for friends and best or close friends only. (C) A network made of best or close friends only.

Figure 1 shows the reconstructed social network of students and its degree distribution at three different levels (A: acquaintance and above, B: friend and above, C: best or close friend only; directions of links are omitted for clarity). At all levels, there were no disconnected nodes or components. As seen in Figure 1A, the network was very dense if all the acquaintances were taken into account, because the students knew almost everybody in the same class. In contrast, the friendship (1B) and best/close friendship (1C) networks had non-trivial structures. Some community structures were also revealed in the best/close friendship network (Figure 1C).

We conducted a linear regression analysis to model the relationships between a student’s academic progress (***y***) and several independent variables, including the student’s original academic ranking and the social environment surrounding the student). The analysis produced the following model equation (also see Table 1):
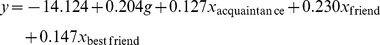



**Table 1:** Result of linear regression of the student’s academic progress over one year (*y*) on four independent variables measured in January 2011.

	*DF*	*SS*	*MS*	*F*-statistic	*p*-value
***g***	1	9011.83	9011.83	12.7903	0.000467***
***x***_acquaintance_	1	1877.14	1877.14	2.6642	0.104686
***x***_friend_	1	4481.44	4481.44	6.3604	0.012692*
***x***_best friend_	1	2785.15	2785.15	3.9529	0.048574*
Error	153	107801.	704.58		
Total	157	125956.			

*y*  =  −14.124 + 0.204*g* + 0.127*x*_acquaintance_ + 0.230*x*_friend_ + 0.147*x*_best friend_

Independent variables are: ***g***: student’s academic ranking, and ***x*** *_i_*: difference in ranking between each student’s self-reported neighbors’ average GPA and his/her own GPA, where ***i*** is either acquaintance, friend or best friend. A highly significant effect of ***g*** was due to the fact that top-ranked students did not change their academic rankings much after one year.

**Figure 2:**
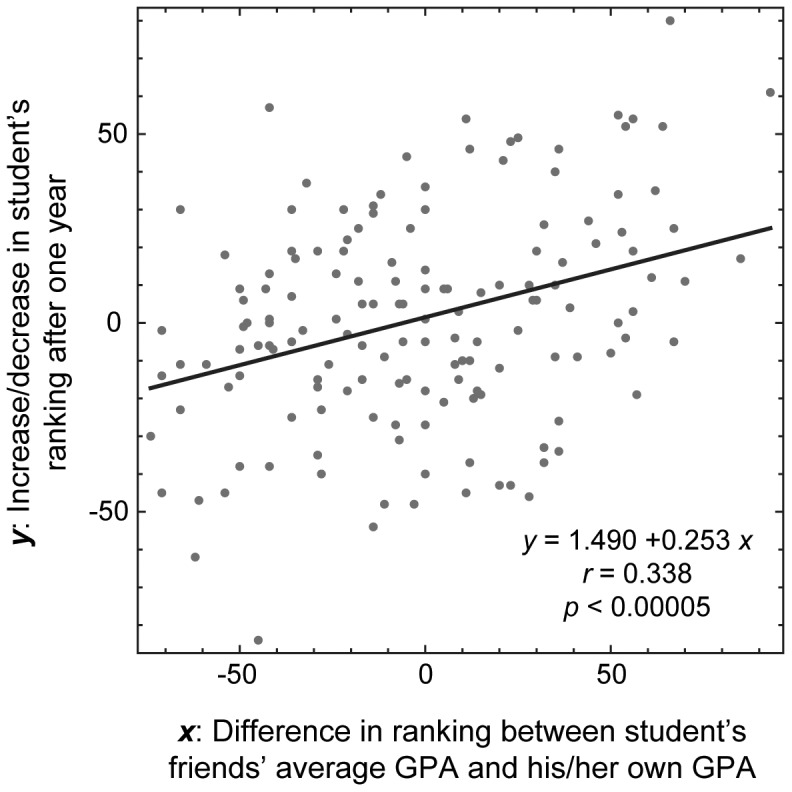
Positive correlation between the social network environment and the student’s academic progress over one year. The horizontal axis represents the difference in ranking between a student’s friends’ average GPA and his/her own GPA (***x*** = ***x***_friend_). The vertical axis represents the increase/decrease in the student’s ranking after one year (***y***). Each dot represents data from one student.

Here ***g*** is the student’s original academic ranking, which had a highly significant effect on ***y*** but this was due to the fact that top-ranked students did not change their academic rankings much after one year. Among the three types of ***x***, the friends (***x***_friend_) had the most significant effect on ***y*** (*p* = 0.0127). Figure 2 is a scatter plot of ***x***_friend_ and ***y***, showing a clear positive correlation between these two variables.

However, we note that ***x*** and ***y*** must be inherently correlated due to their definitions. Namely, a large (or small) value of ***x*** means that the student is likely ranked low (or high) in the class and thus tends to have a large (or small) room for improvement over the following year, naturally causing positive correlation between ***x*** and ***y*** even without any social interactions. To determine whether there was any social influence in effect in the students’ network, we need to compare the observed result with hypothetical null models where social network structures are randomized and neutralized. If the ups and downs of the students’ grades are simply due to individual students’ independent fluctuations, then the observed correlation should not differ from correlations seen in randomized networks.

We therefore conducted a Monte Carlo simulation by randomizing the network topologies and calculating Pearson’s correlation coefficients between ***x***_friend_ and ***y*** for 500 independent simulated cases. In so doing, we kept the number of outgoing links of each student fixed, in order to preserve the out-degree distributions captured in the survey data. This simulates a hypothetical scenario where each student randomly identified a certain number of other students as his or her friends. Similar network randomization techniques that preserve global network topology have been proposed and used extensively in network science [7], [8]. Our assumption in conducing this simulation is that, if the neighbors’ academic rankings had actual social influence on the student’s academic progress, then the correlation coefficient observed in Figure 2 should be significantly higher than its randomized counterparts.

**Figure 3:**
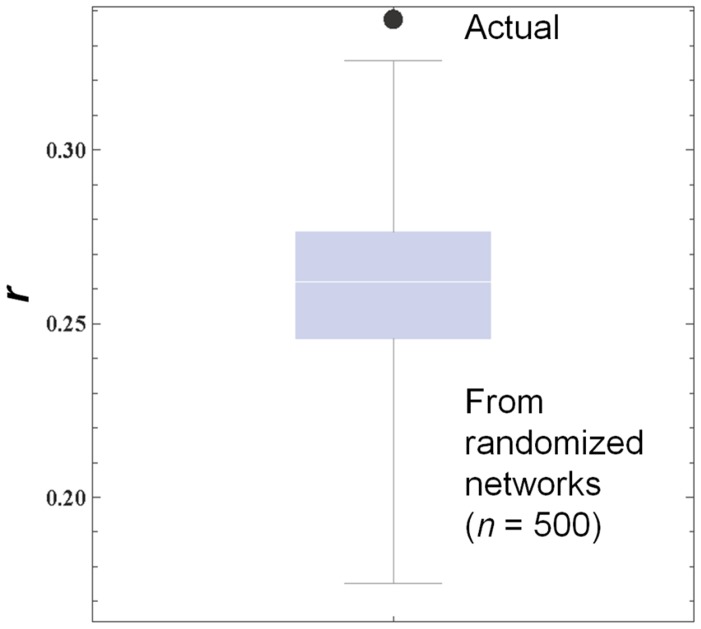
Comparison of Pearson’s correlation coefficients (*r*) between actual and simulated cases. A large dot shows the actual correlation coefficient observed in Figure 2, while the box-whisker plot shows the distribution of simulated correlation coefficients calculated on randomized network topologies in which the out-degrees of individual nodes were all fixed.

Figure 3 compares the actual correlation coefficient with the distribution of simulated correlation coefficients generated from 500 randomized networks. The actual correlation coefficient was larger than the largest simulated coefficient, indicating that the neighbors’ average GPA had a true correlation with the student’s academic progress over the following year.

## Discussion

The results presented above have some important implications. Firstly, the positive correlation between the neighbors’ average GPA and the student’s academic progress indicates that social contagion of academic success may be taking place in the students’ social network, similar to those reported on obesity [9], emotions [10] and other cognitive or behavioral traits [11], [12]. While most educators already know from their experience the importance of social environment for a student’s academic success, our study presents the first quantitative supporting evidence for such empirical knowledge, especially from a social network viewpoint.

Secondly, the regression analysis revealed that the correlation was most significant at the friendship level (Table 1), while the correlations at acquaintance and best/close friendship levels were not as significant. This may be understood in that students tend to choose their best or close friends primarily using a homophilic mechanism on personalities, interests, tastes, favorites, and so on, so the interaction with them would not cause much changes to the student (and also that acquaintances would not cause much changes either, obviously). This finding, that an intermediate level of friendship has the highest influence on an individual, has an interesting similarity to Granovetter’s well-known ‘weak tie’ observation [13].

Thirdly, our research suggests the possibility of a quick test to predict a student’s academic progress that does not require large-scale surveys or complicated social network analysis. The key information used is a student’s self-reported friends’ average GPA relative to the student’s own GPA. This information can be easily collected from a single individual student. One could ask a student who are his/her friends in the class, and test if those self-reported friends’ average GPA is higher or lower than the student’s. If our finding is validated through more extensive studies, this test might serve as a simple, handy predictor of the student’s future performance in various educational settings.

We note that our work is still limited in several aspects. First, the size of the subject population was very small (*N* = 158). It would be desirable to replicate the same study at different schools to test the robustness and generalizability of our observations. Second, the students’ background did not involve much cultural or ethnic diversity. Our subjects were predominantly white, living in a suburban or rural area in Upstate New York. We did not have data to control potential confounding effects of those socio-cultural variables. Lastly, the students’ social network was reconstructed using their self-report responses to the survey, without any other more objective or observational data (e.g., email or text exchanges, physical contacts). We currently do not have capability or resource to collect such data, but recent social science research [14], [15] has demonstrated that emerging technologies are enabling researchers to capture more detailed social dynamics of students at school. It will be very interesting to compare such high-resolution social network data with official school records and analyze correlations between the students’ socio-behavioral patterns and their academic trajectories.
